# Phase I study of metformin in combination with carboplatin/paclitaxel chemotherapy in patients with advanced epithelial ovarian cancer

**DOI:** 10.1007/s10637-020-00920-7

**Published:** 2020-03-07

**Authors:** K. Esther Broekman, Marieke A. J. Hof, Daan J. Touw, Jourik A. Gietema, Hans W. Nijman, Joop D. Lefrandt, An K. L. Reyners, Mathilde Jalving

**Affiliations:** 1 Department of Medical Oncology, University Medical Center Groningen, University of Groningen, PO Box 30.001, 9700 RB Groningen, the Netherlands; 2 Department of Clinical Pharmacy and Pharmacology, University Medical Center Groningen, University of Groningen, Groningen, the Netherlands; 3 Department of Obstetrics and Gynecology, University Medical Center Groningen, University of Groningen, Groningen, the Netherlands; 4 Department of Internal Medicine, University Medical Center Groningen, University of Groningen, Groningen, the Netherlands

**Keywords:** Metformin, Ovarian cancer, Chemotherapy, Safety

## Abstract

*Background* Metformin use is associated with reduced cancer risk in epidemiological studies and has preclinical anti-cancer activity in ovarian cancer models. The primary objective of this phase I study was to determine the recommended phase II dose (RP2D) of metformin in combination with carboplatin/paclitaxel in patients with ovarian cancer. Secondary objectives were to describe safety and pharmacokinetics. *Methods* In this single-center trial the RP2D of metformin in combination with carboplatin area under the concentration-time curve (AUC) 6 and paclitaxel 175 mg/m^2^ every 3 weeks (q3w) in patients with advanced epithelial ovarian cancer was determined using a 3 + 3 escalation rule at three fixed dose levels: 500 mg three times daily (tds), 850 mg tds and 1000 mg tds. Metformin was commenced on day 3 of cycle 1 and continued until 3 weeks after the last chemotherapy administration. The RP2D was defined as the dose level at which 0 of 3 or ≤ 1 of 6 evaluable subjects experienced a metformin-related dose-limiting toxicity (DLT). Safety was assessed according to CTCAE v4.0. Plasma and serum samples for pharmacokinetic (PK) analyses were collected during treatment cycles 1 and 2. *Results* Fifteen patients with epithelial ovarian cancer and an indication for neo-adjuvant (*n* = 5) or palliative (*n* = 10) treatment were included. No DLTs were observed. Three patients discontinued study treatment during cycle 1 for other reasons than DLT. Six patients were treated at the RP2D of metformin 1000 mg tds. The most frequent low-grade toxicities were anemia, hypomagnesemia and diarrhea. Grade 3 adverse events (AEs) occurred in ten patients, most common were leucopenia (*n* = 4), thrombocytopenia (*n* = 3) and increased GGT (*n* = 3). There were no grade 4 AEs. Metformin increased the platinum (Pt) AUC (Δ22%, *p* = 0.013) and decreased the Pt clearance (Δ-28%, *p* = 0.013). Metformin plasma levels were all within the therapeutic range for diabetic patients (0.1–4 mg/L). *Conclusion* The RP2D of metformin in combination with carboplatin and paclitaxel in advanced ovarian cancer is 1000 mg tds. This is higher than the RP2D reported for combination with targeted agents. A potential PK interaction of metformin with carboplatin was identified.

**Electronic supplementary material:**

The online version of this article (10.1007/s10637-020-00920-7) contains supplementary material, which is available to authorized users.

## Introduction

Epithelial ovarian cancer has the highest mortality of all gynecological cancers [1]. The disease is often diagnosed at a late stage because symptoms only develop once the disease has spread throughout the abdominal cavity. First line therapy for advanced disease consists of complete debulking surgery in combination with platinum-based chemotherapy [2]. Initial complete response to therapy can be achieved. However, the majority of patients ultimately develop recurrent disease, with over 50% of women diagnosed with epithelial ovarian cancer eventually dying of their disease [3]. Novel targets are needed in ovarian cancer and targeting metabolic reprogramming is of interest [4, 5]. Mutations in the key tumor suppressor gene and metabolic regulator p53 are the most frequent genetic abnormality occurring in ovarian cancer [6–8]. Furthermore, the mammalian target of rapamycin (mTOR) pathway is overactivated in approximately 70% of ovarian cancers and regulates protein translation of cell growth regulators such as cyclin D1, hypoxia inducible factor 1α (HIF1α) and MYC, all essential for survival under cellular stress [9–11].

Metformin is a biguanide widely used in patients with type 2 diabetes mellitus (T2DM). It improves glycemic control by decreasing insulin resistance, reducing hepatic gluconeogenesis and inhibiting gastro-intestinal glucose resorption [12]. In cancer cells, metformin inhibits mTOR through activation of AMP-activated protein kinase (AMPK) resulting in reduced cellular proliferation [13]. In addition, metformin reduces cellular respiration by inhibiting complex 1 of the mitochondrial respiratory chain limiting the cancer cell’s metabolic plasticity [14–16]. Metformin-treated cancer cells compensate for suppression of oxidative phosphorylation by enhancing glycolysis. This metabolic conversion appears to be p53-dependent [17]. Therefore, in the absence of functional p53, as is usually the case in high grade serous ovarian cancer (HGSOC), cancer cells might be unable to compensate for metformin-induced suppression of oxidative metabolism [18]. In ovarian cancer cell lines and xenograft models, metformin treatment indeed inhibits cell growth, induces apoptosis, inhibits angiogenesis and metastatic spread, potentiates effectivity of platinum treatment and reverses chemotherapy resistance [19–24]. Metformin use has been associated with a reduced ovarian cancer risk and reduced (ovarian) cancer-specific mortality in diabetic patients, compared to non-use and use of other hypoglycemic drugs [25–27].

Taken together, the body of preclinical and epidemiological evidence suggests a potential role for metformin in the treatment of advanced ovarian cancer, especially in combination with carboplatin [28]. Therefore, we conducted a phase I dose-escalation trial to establish the recommended phase II dose (RP2D) for further evaluation of the effects of metformin treatment in combination with carboplatin in advanced ovarian cancer.

## Methods

### Patient population

Adult patients with advanced stage (FIGO III-IV), histologically confirmed epithelial ovarian cancer eligible for carboplatin/paclitaxel chemotherapy, either in the neo-adjuvant or palliative setting, were eligible for this study. Patients were required to have an ECOG-performance status of 0–2, adequate blood counts, hepatic and renal function (eGFR ≥60 ml/min, calculated by the Cockcroft-Gault equation) and provide written informed consent for trial participation. Patients that had used metformin within 4 weeks of study enrolment were excluded, as were patients with a known hypersensitivity to the study drugs. No patients with diabetes mellitus were included. Patients with symptomatic central nervous system (CNS) metastases or peripheral neuropathy ≥ CTCAE v4.0 grade 2, serious active infections or other unstable medical conditions were also excluded. Detailed in- and exclusion criteria are listed in Supplementary Table 1.

### Study design

A phase I dose-escalation trial was performed in a single tertiary center in the Netherlands. In addition to standard carboplatin/paclitaxel chemotherapy, metformin was administered in escalating doses according to a 3 + 3 design. Three patients were included per metformin dose level cohort (Table 1). In case any of these patients experienced a DLT, the cohort would be expanded to six patients. If ≤1 of these six patients experienced a DLT, escalation to the next dose level was permitted. The maximum administered dose (MAD) was defined as the dose level at which ≥2 patients experienced a DLT. The RP2D was defined as one dose level below the MAD. If the MAD was reached at the starting dose level, three to six patients would be treated at dose level ‘−1’ and in absence of DLT this would be the RP2D. In case no DLT was seen at the highest planned dose level this would be the RP2D. Additional patients were recruited into the recommended phase II dose level to include at least six patients. Safety was assessed for all patients entering the trial and receiving at least one dose of metformin. Toxicity was graded using CTCAE v4.0 [29], and dose-limiting toxicities were assessed until the end of cycle 2. A DLT was defined as a toxicity related to metformin, fulfilling one of the criteria in Supplementary Table 2. Any AE that occurred after start of study treatment or worsened during study treatment was defined as a treatment-emergent adverse event (TEAE).

**Table 1:** Dose levels and dose-escalation schedule for metformin

Dose level	Dose of metformin given orally (total daily dose)	Minimum number of patients
-1	500 mg bd (1000 mg)	(3, if DLT at level 1)
1	500 mg tds (1500 mg)	3
2	850 mg tds (2250 mg)	3
3	1000 mg tds (3000 mg)	3

BD two times daily, DLT dose-limiting toxicity, TDS three times daily

### Study treatment

Patients received carboplatin AUC = 6 and paclitaxel 175 mg/m^2^ as a 3-h infusion on day 1 of every 3-week cycle. A maximum of 6 cycles were administered, with the exception of patients whose CA-125 had not normalized but was still declining during cycle 6 of palliative treatment. These patients were permitted a maximum of 3 extra cycles. Dose modifications or delays of carboplatin and paclitaxel chemotherapy were executed according to standard clinical practice. Metformin was commenced on day 3 of cycle 1, to allow for pharmacokinetic (PK) analyses of carboplatin and paclitaxel in the absence of metformin. Within every dose level cohort, the dose of metformin was gradually increased during cycle 1, starting at 500 mg bd and increasing by one dose level every 3 days to the target dose level (Table 1). Intra-patient dose-escalation of metformin results in better tolerance in diabetic patients [30, 31]. The highest planned metformin dose level was 1000 mg tds, corresponding to the maximum recommended dose for treatment of T2DM [30]. Metformin treatment was discontinued 3 days before a planned CT-scan or surgery and resumed afterwards using the aforementioned dose-escalation scheme. CT-scans and measurements of ca-125 for evaluation of response to treatment were performed according to standard practice.

### Pharmacokinetic analysis

To assess the influence of metformin on the PK parameters of carboplatin and paclitaxel, these were evaluated in cycle 1 (without metformin) and cycle 2 (with metformin). To determine the complete concentration-time curve, a 9-sample design was used for carboplatin for which plasma samples were drawn after 30 min, 1 h, 1.5 h, 2 h, 3.5 h, 5.5 h, 9.5 h, 24 h (± 2 h) and 72 h (± 2 h) after administration of carboplatin. For paclitaxel, four of these time points were used, namely 30 min, 3.5 h, 24 h (± 2 h) and 72 h (± 2 h) after paclitaxel administration. The following parameters of carboplatin and paclitaxel PK were compared using the PK modeling software MWPharm version 3.82 (Mediware, Groningen, Netherlands): maximum concentration (Cmax), time to reach maximum concentration (Tmax), AUC, half-life (t1/2), total body clearance and apparent volume of distribution (Vd).

Metformin was analyzed at the Department of Clinical Pharmacy and Pharmacology of the University Medical Center Groningen using a validated liquid chromatography-tandem mass spectometry (LC-MS/MS) method. Samples were diluted using a solution containing 2H-labeled metformin. The lower limit of quantitation (LLOQ) was 0.1 mg/L and the upper limit of quantitation (ULOQ) was 100 mg/L. The coefficient of variation was less than 4.4% over the entire working range. Platinum (Pt) was analyzed using a validated ICP-MS method. The LLOQ was 1 microg/L and the ULOQ was 3000 microg/L. The coefficient of variation was less than 4.7% over the entire working range. Paclitaxel was analyzed at the Netherlands Cancer Institute Amsterdam using a validated LC-MS/MS method [32]. The concentration range was between 0.5 and 500 microg/L. Samples with concentrations above the ULOQ were diluted and reanalyzed. Inter-assay accuracy and precision were tested at four concentration levels and were within 10% and less than 10%, respectively. All analytical methods were compliant with the EMA guidelines for bio-analytical method validation (https://www.ema.europa.eu/en/bioanalytical-method-validation). PK profiles of carboplatin and paclitaxel were determined during cycle 1 and cycle 2. The AUC was calculated until the last time-point measured. Microsoft Excel 2010 was used to carry out the numerical integration (Δt = 1 min) to calculate the AUC. Pt levels during and after infusion were calculated according to a 3-compartment PK model using the KinFit module in MWPharm [33].

### Statistical analysis

Baseline characteristics and incidence of AEs were summarized using descriptive statistics. For the PK analyses, intra-patient comparisons were performed using the Wilcoxon signed rank test for AUC, Vd and clearance of carboplatin and paclitaxel. All *p* values were calculated using IBM SPSS Statistics for Windows version 23, assuming non-parametric distribution. *P* < 0.05 was considered to indicate a statistically significant difference.

## Results

### Characteristics of the study cohort

**Fig. 1:**
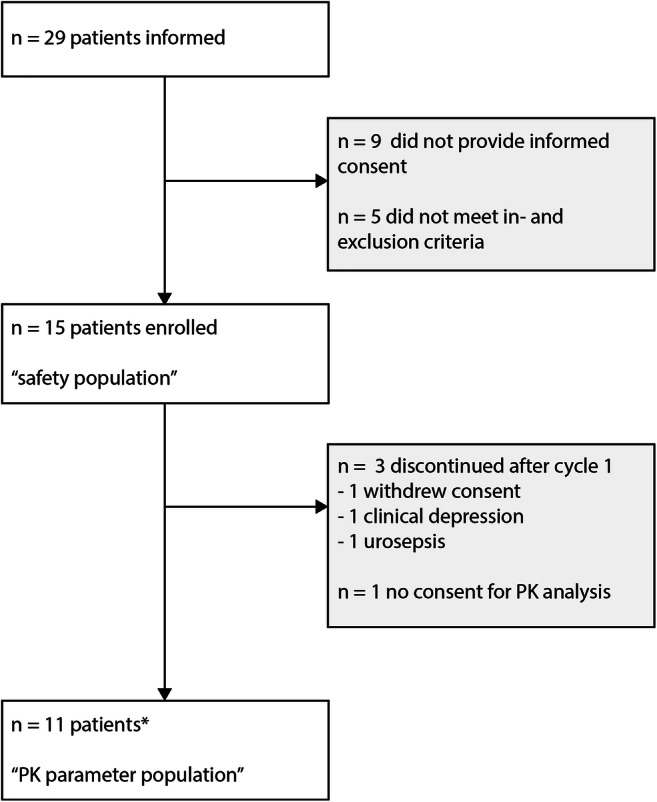
Consort diagram of patient flow through the study. * For one patient plasma samples were not drawn at the correct times to allow for a valid Pt AUC calculation. PK of carboplatin was therefore assessed in 10 patients.

29 patients were informed about the trial. Nine patients declined and five did not meet the in- and exclusion criteria. Fifteen eligible patients were enrolled in the trial and received ≥1 dose of metformin (*n* = 5 neo-adjuvant and *n* = 10 palliative setting), this population was used for the safety assessment (Fig. 1). The baseline characteristics of these 15 patients are shown in Table 2. Three patients discontinued study treatment during cycle 1 for other reasons than DLT: one patient withdrew consent, and two patients experienced a serious AE (SAE) unrelated to metformin treatment but resulting in study discontinuation (one patient with a serious depression and one patient with a urosepsis). One patient did not provide consent for PK-analysis and for one patient plasma samples were not drawn at the correct times to allow a valid Pt AUC calculation. The population for PK analysis of carboplatin and paclitaxel therefore consisted of the 10 and 11 patients respectively for whom valid and evaluable PK parameters were derived during cycle 1 and cycle 2 of treatment.

**Table 2:** Baseline characteristics of study participants

Baseline characteristics (*n* = 15)	
Age (years)	
Median (range)	66 (55–71)
Setting, N (%)	
Neo-adjuvant	5 (33)
Palliative	10 (67)
FIGO stage, N (%)	
IIIC	7 (47)
IV	8 (53)
ECOG-PS, N (%)	
0	10 (67)
1	5 (33)
GFR (mL/min, Cockcroft-Gault), median (range)	84 (60–120)
Previous chemotherapy lines, N (%)	
0	5 (33)
1	4 (27)
2	3 (20)
>2	3 (20)

ECOG-PS Eastern Cooperative Oncology Group Performance Status*,* FIGO Fédération Internationale de Gynécologie Obstétrique*,* GFR glomerular filtration rate

### MTD and DLTs

No DLTs were observed for metformin. Six patients were treated at the 1000 mg tds metformin dose level. Safety data showed that most common low-grade toxicities were anemia, hypomagnesemia and diarrhea. Grade 3 treatment-emergent adverse events (TEAEs) occurred in nine patients, most commonly leucopenia (*n* = 4), thrombocytopenia (*n* = 3) and increased GGT (*n* = 2). There were no grade 4 TEAEs. All grade 3 TEAEs and grade 1–2 TEAEs occurring in ≥10% of patients are listed in Table 3.

**Table 3:** Treatment emergent adverse events

Adverse events, N (%) (*n* = 15) CTCAEv4.0 grade (worst per patient)	1–2	3
Anemia	12 (80)	1 (7)
Leucopenia	8 (53)	4 (27)
Thrombocytopenia	6 (40)	3 (20)
Diarrhea	8 (53)	
Nausea	5 (33)	
Vomiting	2 (13)	
Abdominal pain	2 (13)	
Ascites	1 (7)	1 (7)
Hypomagnesemia	11 (73)	1 (7)
Hyponatremia	2 (13)	1 (7)
Increased ALT	8 (53)	
Increased AST	5 (33)	
Increased GGT	3 (20)	2 (13)
Increased creatinine	2 (13)	
Dyspnea		1 (7)
Neuropathy	6 (40)	
Fatigue	4 (27)	
Extremity pain	3 (20)	
Urinary tract infection		1 (7)

CTCAE common terminology criteria for adverse events

Twelve patients completed all planned carboplatin cycles. Three patients stopped paclitaxel treatment early; one patient after cycle 4 due to myelosuppression and two patients did not receive the last paclitaxel cycle due to neuropathy. In three patients the carboplatin dose was reduced due to thrombocytopenia, in one patient the dose was reduced by two levels. Paclitaxel dose was reduced by one level in eight patients, due to chemotherapy-related myelosuppression (*n* = 3) or neuropathy (*n* = 5). Seven patients required one or more dose delays (one *n* = 2; two *n* = 3; four *n* = 2). Dose delays were due to chemotherapy-related hematological toxicity in six patients and a viral infection in one patient.

### Pharmacokinetics

**Fig. 2:**
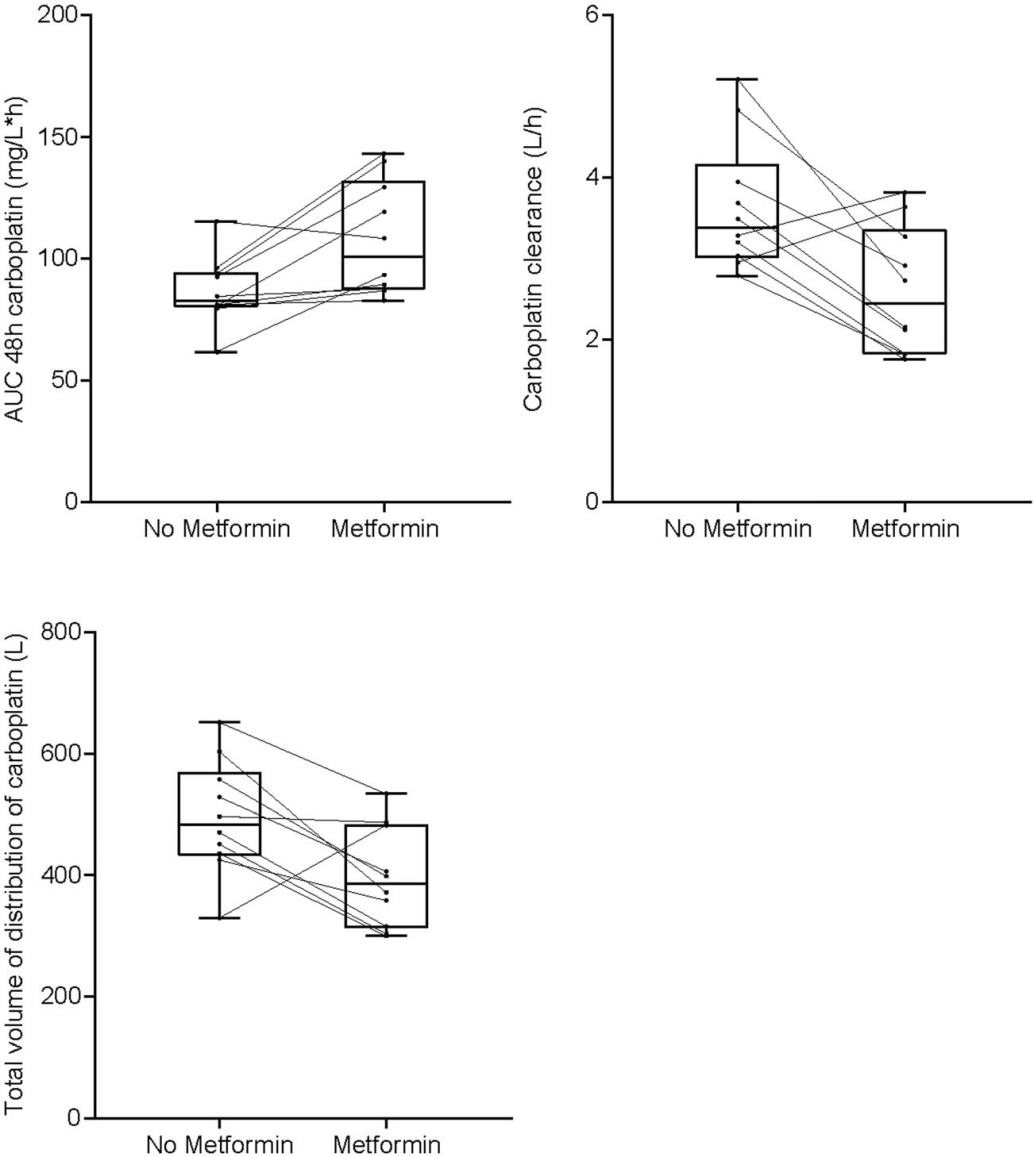
Platinum AUC, clearance and total volume of distribution without (cycle 1) and with metformin (cycle 2), calculated from measurement of platinum in plasma samples in 10 patients in three dose cohorts of metformin (metformin 500 mg bd *n* = 2, 850 mg tds *n* = 3, 1000 mg tds *n* = 5). The lines between the two boxplots indicate the intra-individual changes of platinum AUC, clearance and total volume of distribution (*n* = 10 patients). Platinum AUCs of cycle 2 were corrected for carboplatin dose adjustment from cycle 1 to cycle 2 in 3 patients (dose reduction for reason of thrombocytopenia), assuming a linear relationship between dose and AUC

**Fig. 3:**
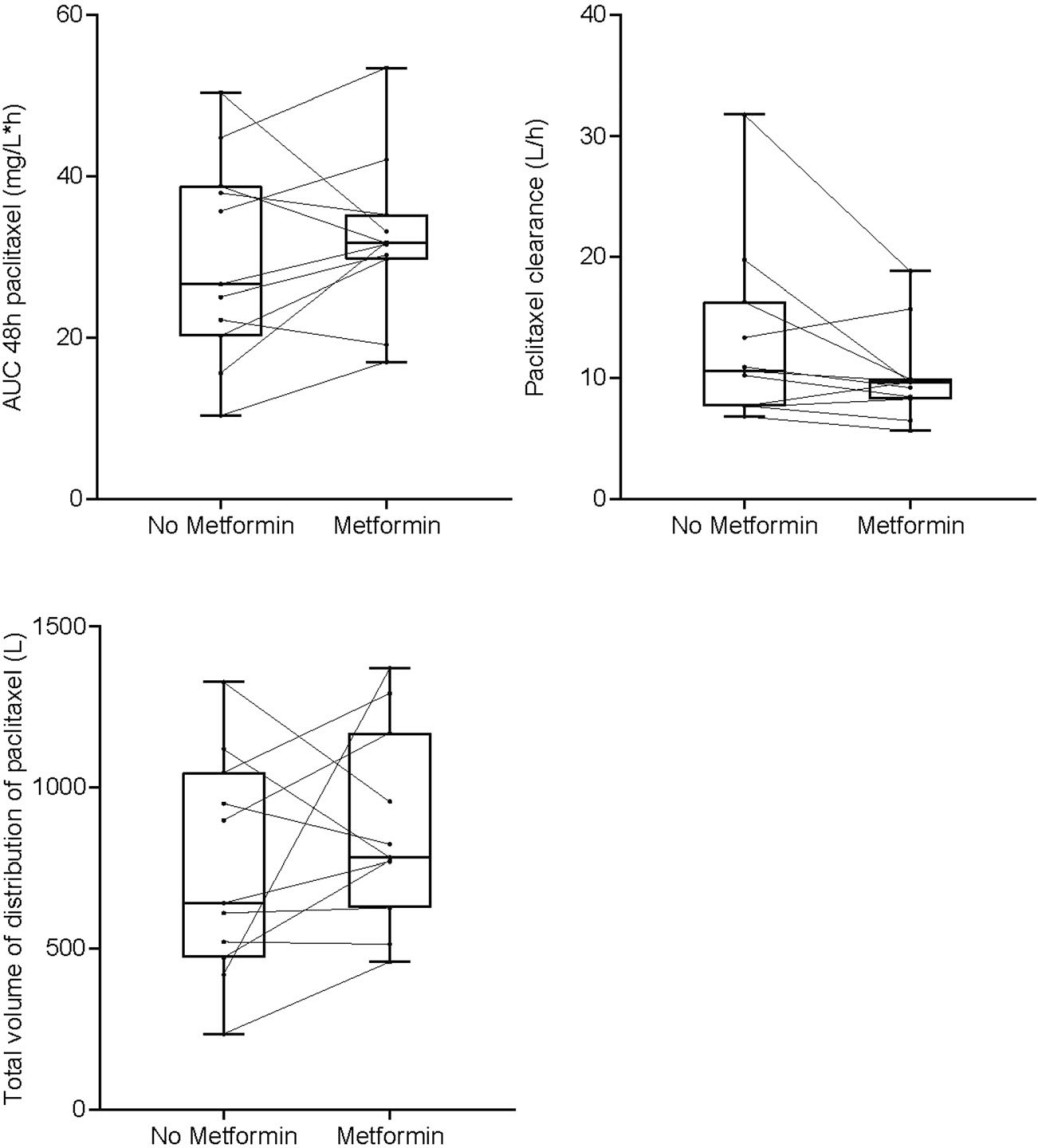
Paclitaxel AUC, clearance and total volume of distribution without (cycle 1) and with metformin (cycle 2), calculated from measurement of paclitaxel in plasma samples in 11 patients in three dose cohorts of metformin (metformin 500 mg bd *n* = 3, 850 mg tds n = 3, 1000 mg tds *n* = 5). The lines between the two boxplots indicate the intra-individual changes of carboplatin AUC, clearance and total volume of distribution (*n* = 11 patients). Paclitaxel AUC of cycle 2 was corrected for paclitaxel dose adjustment from cycle 1 to cycle 2 in 1 patient (dose reduction for reason of leukopenia), assuming a linear relationship between dose and AUC

Platinum PK analyses were performed in 10 patients. The AUC was calculated for t = 0 to t = 48 h, because in two patients the 72 h sample was missing. Median PK parameters of Pt are shown in Table 4. Carboplatin AUCs in cycle 2 were corrected for carboplatin dose adjustment from cycle 1 to cycle 2 in three patients (dose reduction due to thrombocytopenia), assuming a linear relationship between dose and AUC, to assess the effect of metformin on Pt AUC. PK analysis showed a metformin induced increase in the AUC of Pt (82.8 mg/L*h vs. 101 mg/L*h, Δ 22.0%, *p* = 0.013) and a decrease in the Pt clearance (3.39 L/h vs. 2.44 L/h, Δ-28%, *p* = 0.013). There were no differences in Pt AUC 48 h between dose groups; however the small group sizes hamper a firm conclusion. A decrease of 20.5% in median total volume of distribution (Vd) of Pt was found when co-administrated with metformin (484 L vs.385 L, *p* = 0.037) (Fig. 2). When patients were ranked based on carboplatin AUC and clearance, there was no correlation between these parameters and occurrence of known carboplatin toxicities such as thrombocytopenia and hypomagnesemia. Moreover, there was no correlation between metformin induced changes in carboplatin pharmacokinetics and toxicity. There were no differences in paclitaxel AUC, clearance or Vd between cycle 1 without metformin and cycle 2 of treatment with metformin (Fig. 3, Supplementary Table 3). All measured metformin levels were within the therapeutic range of 0.1–4 mg/L for diabetic patients) [34, 35].

**Table 4:** Pharmacokinetic parameters of platinum

Parameter	Unit	Carboplatin without metformin (median (IQR))	Carboplatin with metformin (median (IQR))	*p* value
CL	L/h	3.39 (1.2)	2.44 (1.5)	0.013
Vd^a^	L	484 (135.6)	385 (170.9)	0.037
AUC 48 h	mg/L*h	82.8 (14.2)	91.8 (35.0)	0.028
AUC 48 h corrected for dose adjustment	mg/L*h	82.8 (14.2)	101 (44.7)	0.013

Pharmacokinetic parameters of platinum (carboplatin) when administered as combination chemotherapy with paclitaxel and with addition of metformin (*n* = 10 patients)

^a^With the KinFit analysis, it was determined that a three-compartment model fit the platinum levels best based on the improved *p*-values of the fits compared to a two-compartment model. No significant difference in volume of distribution was found in any of the three separate compartments.

Platinum AUCs of cycle 2 were corrected for carboplatin dose adjustment from cycle 1 to cycle 2 in 3 patients (dose reduction for reason of thrombocytopenia), assuming a linear relationship between dose and AUC. AUC area under the concentration-time curve, CL clearance, IQR inter quartile range, Vd total volume of distribution

### Evaluation of response to treatment

Response assessment was not an endpoint in this study and was performed according to standard clinical practice. Treatment efficacy parameters for individual patients are listed in Supplementary Table 4.

## Discussion

The recommended phase II dose (RP2D) of metformin in combination with carboplatin and paclitaxel in our study was 1000 mg tds (3000 mg/day).

This RP2D is higher than in other early phase trials where metformin was combined with other anti-cancer therapy. Overlapping gastro-intestinal toxicities of metformin with, for example, mTOR-inhibitors and 5-FU based chemotherapy may have led to lower tolerability of combination therapy in these trials. Furthermore, relatively fit patients were included in our study; 67% had an ECOG-PS of 0 and 33% an ECOG-PS of 1. Metformin tolerability as monotherapy and in combination with anti-cancer therapy is improved when intra-patient dose-escalation schemes are used [36–39]. The dose-escalation scheme used in our study, likely contributed to the relatively high RP2D (3000 mg/day). The characteristics of previous trials can be found in supplementary Table 5 [36–48].

The combination of metformin with carboplatin and paclitaxel was tolerable and no dose-limiting toxicities occurred. Diarrhea is a well-known side effect of metformin, occurring in 20% of metformin treated diabetic patients leading to discontinuation in 5% of patients [49, 50]. Diarrhea occurred in eight patients in our study (53%), all grade 1–2. Although this is comparable to rates observed in other metformin combination studies it must be considered that diarrhea, even at low grades, can have a serious impact on health-related quality of life, and reduce treatment adherence. Hypomagnesemia is reported in 18% of type 2 diabetes mellitus patients receiving metformin [51], and even more frequently in patients receiving carboplatin (up to 46%) [52]. We therefore expected to observe an increased frequency of hypomagnesemia. Hypomagnesemia indeed occurred in 12 of 15 patients (80%) in our trial. However, in the majority of patients this was mild (11 patients grade 1–2) and all patients were treated with oral supplementation only. Other frequently occurring adverse events were related to myelosuppression, with frequencies not higher than expected for carboplatin and paclitaxel chemotherapy.

Metformin increased the Pt AUC by 22% and reduced Pt clearance by28%. Due to the small number of patients per dose group, it was not possible to determine whether metformin had a dose-dependent effect on Pt AUC and clearance. There were no other obvious factors explaining the difference in Pt AUC and Pt clearance between cycle 1 and cycle 2. GFR did not change significantly, changes in albumin levels were not considered to be of influence because carboplatin does not bind to protein in vitro [53] and corrections for body weight changes were incorporated in the calculation of the carboplatin doses in cycle 1 and cycle 2.

The metformin-induced reduction in renal clearance of Pt could be due to a direct effect of metformin on the tubular resorption of Pt, via its copper-binding properties [54, 55]. Copper transporters 1 and 2 (CTR1 and CTR2) regulate both copper-transport and transport of carboplatin as well as cisplatin into the cell [56, 57]. Copper-deficiency can be induced by metformin binding to copper which may result in upregulation of CTR1 as a compensatory mechanism [58]. Upregulation of CTR1 in kidney tubular cells may result in improved retention of carboplatin resulting in decreased clearance. Intracellular copper deficiency in cancer cells may also enhance carboplatin uptake due to upregulation of copper transporters [59]. Five patients with platinum resistant HGSOC were treated with carboplatin in combination with the copper chelator trientine yielding a partial response in one patient and stable disease in three patients [60]. Metformin has indeed been shown to reverse platinum-resistance of ovarian cancer cells through various mechanisms, but copper transporters have not been evaluated in these models [61, 62]. The majority of preclinical studies on anti-cancer effects of metformin used higher concentrations of metformin than those that can be achieved in patients with diabetes [63]. However, metformin has been shown to accumulate in tumor tissue, potentially due to the acidic tumor microenvironment, which could well result in intratumoral concentrations in the ranges studied preclinically [64, 65].

In conclusion, our study showed that the addition of metformin to carboplatin/paclitaxel in advanced ovarian cancer is feasible and well tolerated, with a RP2D of 1000 mg tds. A potential PK interaction of metformin with carboplatin was identified.

## Electronic supplementary material

ESM 1 (DOCX 25 kb)

## References

[1] Siegel RL, Miller KD, Jemal A (2019). Cancer statistics, 2019. CA Cancer J Clin.

[2] Vergote I, Tropé CG, Amant F, Kristensen GB, Ehlen T, Johnson N, Verheijen RHM, van der Burg MEL, Lacave AJ, Panici PB, Kenter GG, Casado A, Mendiola C, Coens C, Verleye L, Stuart GCE, Pecorelli S, Reed NS (2010). Neoadjuvant chemotherapy or primary surgery in stage IIIC or IV ovarian cancer. N Engl J Med.

[3] Torre LA, Trabert B, DeSantis CE, Miller KD, Samimi G, Runowicz CD, Gaudet MM, Jemal A, Siegel RL (2018). Ovarian cancer statistics, 2018. CA Cancer J Clin.

[4] Cancer Genome Atlas Research Network (2011). Integrated genomic analyses of ovarian carcinoma. Nature.

[5] Han CY, Patten DA, Richardson RB, Harper ME, Tsang BK (2018). Tumor metabolism regulating chemosensitivity in ovarian cancer. Genes Cancer.

[6] ÓhAinmhire E, Quartuccio SM, Cheng W, Ahmed RA, King SM, Burdette JE (2014). Mutation or loss of p53 differentially modifies TGFβ action in ovarian cancer. PLoS One.

[7] Hall J, Paul J, Brown R (2004). Critical evaluation of p53 as a prognostic marker in ovarian cancer. Expert Rev Mol Med.

[8] Vousden KH, Ryan KM (2009). P53 and metabolism. Nat Rev Cancer.

[9] Guertin DA, Sabatini DM (2007). Defining the role of mTOR in cancer. Cancer Cell.

[10] Porta C, Paglino C, Mosca A (2014). Targeting PI3K/Akt/mTOR signaling in Cancer. Front Oncol.

[11] Mabuchi S, Kuroda H, Takahashi R, Sasano T (2015). The PI3K/AKT/mTOR pathway as a therapeutic target in ovarian cancer. Gynecol Oncol.

[12] Cusi K, Consoli A, DeFronzo RA (1996). Metabolic effects of metformin on glucose and lactate metabolism in noninsulin-dependent diabetes mellitus. J Clin Endocrinol Metab.

[13] Shaw RJ, Bardeesy N, Manning BD, Lopez L, Kosmatka M, DePinho RA, Cantley LC (2004). The LKB1 tumor suppressor negatively regulates mTOR signaling. Cancer Cell.

[14] Kurelac I, Umesh Ganesh N, Iorio M, Porcelli AM, Gasparre G (2019) The multifaceted effects of metformin on tumor microenvironment. Semin Cell Dev Biol. 10.1016/j.semcdb.2019.05.010 10.1016/j.semcdb.2019.05.010 31091466

[15] Gui DY, Sullivan LB, Luengo A, Hosios AM, Bush LN, Gitego N, Davidson SM, Freinkman E, Thomas CJ, Vander Heiden MG (2016). Environment dictates dependence on mitochondrial complex I for NAD+ and aspartate production and determines cancer cell sensitivity to metformin. Cell Metab.

[16] Luengo A, Sullivan LB, Heiden MG (2014). Understanding the complex-I-ty of metformin action: limiting mitochondrial respiration to improve cancer therapy. BMC Biol.

[17] Buzzai M, Jones RG, Amaravadi RK, Lum JJ, DeBerardinis RJ, Zhao F, Viollet B, Thompson CB (2007). Systemic treatment with the antidiabetic drug metformin selectively impairs p53-deficient tumor cell growth. Cancer Res.

[18] Li X, Li B, Ni Z, Zhou P, Wang B, He J, Xiong H, Yang F, Wu Y, Lyu X, Zhang Y, Zeng Y, Lian J, He F (2017). Metformin synergizes with BCL-XL/BCL-2 inhibitor ABT-263 to induce apoptosis specifically in p53-defective cancer cells. Mol Cancer Ther.

[19] Gotlieb WH, Saumet J, Beauchamp M, Gu J, Lau S, Pollak MN, Bruchim I (2008). In vitro metformin anti-neoplastic activity in epithelial ovarian cancer. Gynecol Oncol.

[20] Rattan R, Graham RP, Maguire JL, Giri S, Shridhar V (2011). Metformin suppresses ovarian cancer growth and metastasis with enhancement of cisplatin cytotoxicity in vivo. Neoplasia.

[21] Rattan R, Giri S, Hartmann LC, Shridhar V (2011). Metformin attenuates ovarian cancer cell growth in an AMP-kinase dispensable manner. J Cell Mol Med.

[22] Yasmeen A, Beauchamp M, Piura E, Segal E, Pollak M, Gotlieb WH (2011). Induction of apoptosis by metformin in epithelial ovarian cancer: involvement of the Bcl-2 family proteins. Gynecol Oncol.

[23] Wu B, Li S, Sheng L, Zhu J, Gu L, Shen H, La D, Hambly BD, Bao S, Di W (2012). Metformin inhibits the development and metastasis of ovarian cancer. Oncol Rep.

[24] Liu Y, Feng Y, Liu H, Wu J, Tang Y, Wang Q (2018). Real-time assessment of platinum sensitivity of primary culture from a patient with ovarian cancer with extensive metastasis and the platinum sensitivity enhancing effect by metformin. Oncol Lett.

[25] Shi J, Liu B, Wang H, Zhang T, Yang L (2019). Association of metformin use with ovarian cancer incidence and prognosis: a systematic review and meta-analysis. Int J Gynecol Cancer.

[26] Romero IL, McCormick A, McEwen KA, Park S, Karrison T, Yamada SD, Pannain S, Lengyel E (2012). Relationship of type II diabetes and metformin use to ovarian cancer progression, survival, and chemosensitivity. Obstet Gynecol.

[27] Wang S, Lei K, Liu J, Jia Y (2017). Continuous use of metformin can improve survival in type 2 diabetic patients with ovarian cancer: a retrospective study. Medicine (Baltimore).

[28] Hajjar J, Habra MA, Naing A (2013). Metformin: an old drug with new potential. Expert Opin Investig Drugs.

[29] CTCAE v4.0 website via https://evs.nci.nih.gov/ftp1/CTCAE/CTCAE_4.03/CTCAE_4.03_2010-06-14_QuickReference_8.5x11.pdf. Accessed 25-11-2019

[30] Metformin Summary of Product Characteristics via http://ema.europa.eu. Accessed 06-03-2019

[31] Nathan DM, Buse JB, Davidson MB, Heine RJ, Holman RR, Sherwin R, Zinman B (2006). Management of hyperglycaemia in type 2 diabetes: a consensus algorithm for the initiation and adjustment of therapy. A consensus statement from the American Diabetes Association and the European Association for the Study of Diabetes. Diabetologia.

[32] Hendrikx JJ, Hillebrand MJ, Thijssen B, Rosing H, Schinkel AH, Schellens JH, Beijnen JH (2011). A sensitive combined assay for the quantification of paclitaxel, docetaxel and ritonavir in human plasma using liquid chromatography coupled with tandem mass spectrometry. J Chromatogr B Analyt Technol Biomed Life Sci.

[33] Proost JH, Meijer DK (1992). MW/pharm, an integrated software package for drug dosage regimen calculation and therapeutic drug monitoring. Comput Biol Med.

[34] Metformin Prescribing Information (2019) via https://www.drugs.com/pro/metformin.html#s-34090-1. Accessed 25-11-2019

[35] Scheen AJ (1996). Clinical pharmacokinetics of metformin. Clin Pharmacokinet.

[36] Khawaja MR, Nick AM, Madhusudanannair V, Fu S, Hong D, McQuinn LM, Ng CS, Piha-Paul SA, Janku F, Subbiah V, Tsimberidou A, Karp D, Meric-Bernstam F, Lu KH, Naing A (2016). Phase I dose escalation study of temsirolimus in combination with metformin in patients with advanced/refractory cancers. Cancer Chemother Pharmacol.

[37] Yam C, Esteva FJ, Patel MM, Raghavendra AS, Ueno NT, Moulder SL, Hess KR, Shroff GS, Hodge S, Koenig KH, Chavez Mac Gregor M, Griner RL, Yeung SJ, Hortobagyi GN, Valero V (2019). Efficacy and safety of the combination of metformin, everolimus and exemestane in overweight and obese postmenopausal patients with metastatic, hormone receptor-positive, HER2-negative breast cancer: a phase II study. Investig New Drugs.

[38] Kordes S, Pollak MN, Zwinderman AH, Mathot RA, Weterman MJ, Beeker A, Punt CJ, Richel DJ, Wilmink JW (2015). Metformin in patients with advanced pancreatic cancer: a double-blind, randomised, placebo-controlled phase 2 trial. Lancet Oncol.

[39] Parikh AB, Kozuch P, Rohs N, Becker DJ, Levy BP (2017). Metformin as a repurposed therapy in advanced non-small cell lung cancer (NSCLC): results of a phase II trial. Investig New Drugs.

[40] Marrone KA, Zhou X, Forde PM, Purtell M, Brahmer JR, Hann CL, Kelly RJ, Coleman B, Gabrielson E, Rosner GL, Ettinger DS (2018). A randomized phase II study of metformin plus paclitaxel/carboplatin/Bevacizumab in patients with chemotherapy-naive advanced or metastatic nonsquamous non-small cell lung Cancer. Oncologist.

[41] Morgillo F, Fasano M, Della Corte CM, Sasso FC, Papaccio F, Viscardi G, Esposito G, Di Liello R, Normanno N, Capuano A, Berrino L, Vicidomini G, Fiorelli A, Santini M, Ciardiello F (2017). Results of the safety run-in part of the METAL (METformin in advanced lung cancer) study: a multicentre, open-label phase I-II study of metformin with erlotinib in second-line therapy of patients with stage IV non-small-cell lung cancer. ESMO Open.

[42] Molenaar RJ, van de Venne T, Weterman MJ, Mathot RA, Klümpen H, Richel DJ, Wilmink JW (2018). A phase Ib study of everolimus combined with metformin for patients with advanced cancer. Investig New Drugs.

[43] Miranda VC, Braghiroli MI, Faria LD, Bariani G, Alex A, Bezerra Neto JE, Capareli FC, Sabbaga J, Lobo Dos Santos JF, Hoff PM, Riechelmann RP (2016). Phase 2 trial of metformin combined with 5-fluorouracil in patients with refractory metastatic colorectal cancer. Clin Color Cancer.

[44] Nanni O, Amadori D, De Censi A, Rocca A, Freschi A, Bologna A, Gianni L, Rosetti F, Amaducci L, Cavanna L, Foca F, Sarti S, Serra P, Valmorri L, Bruzzi P, Corradengo D, Gennari A, MYME investigators (2019). Metformin plus chemotherapy versus chemotherapy alone in the first-line treatment of HER2-negative metastatic breast cancer. The MYME randomized, phase 2 clinical trial. Breast Cancer Res Treat.

[45] Ramos-Penafiel C, Olarte-Carrillo I, Ceron-Maldonado R, Rozen-Fuller E, Kassack-Ipina JJ, Melendez-Mier G, Collazo-Jaloma J, Martinez-Tovar A (2018). Effect of metformin on the survival of patients with ALL who express high levels of the ABCB1 drug resistance gene. J Transl Med.

[46] Trucco M, Barredo JC, Goldberg J, Leclerc GM, Hale GA, Gill J, Setty B, Smith T, Lush R, Lee JK, Reed DR (2018). A phase I window, dose escalating and safety trial of metformin in combination with induction chemotherapy in relapsed refractory acute lymphoblastic leukemia: metformin with induction chemotherapy of vincristine, dexamethasone, PEG-asparaginase, and doxorubicin. Pediatr Blood Cancer.

[47] Reni M, Dugnani E, Cereda S, Belli C, Balzano G, Nicoletti R, Liberati D, Pasquale V, Scavini M, Maggiora P, Sordi V, Lampasona V, Ceraulo D, Di Terlizzi G, Doglioni C, Falconi M, Piemonti L (2016). (Ir)relevance of metformin treatment in patients with metastatic pancreatic Cancer: an open-label, randomized phase II trial. Clin Cancer Res.

[48] Sayed R, Saad AS, El Wakeel L, Elkholy E, Badary O (2015). Metformin addition to chemotherapy in stage IV non-small cell lung Cancer: an open label randomized controlled study. Asian Pac J Cancer Prev.

[49] Bailey CJ, Turner RC (1996). Metformin. N Engl J Med.

[50] Dandona P, Fonseca V, Mier A, Beckett AG (1983). Diarrhea and metformin in a diabetic clinic. Diabetes Care.

[51] Peters KE, Chubb SAP, Davis WA, Davis TME (2013). The relationship between hypomagnesemia, metformin therapy and cardiovascular disease complicating type 2 diabetes: the Fremantle diabetes study. PLoS One.

[52] Herbert C, Cornes P (2011). The unexpected burden of hypomagnesaemia in gynae-oncology chemotherapy clinics. Clin Oncol (R Coll Radiol).

[53] Oguri S, Sakakibara T, Mase H, Shimizu T, Ishikawa K, Kimura K, Smyth RD (1988). Clinical pharmacokinetics of carboplatin. J Clin Pharmacol.

[54] Muller S, Versini A, Sindikubwabo F, Belthier G, Niyomchon S, Pannequin J, Grimaud L, Caneque T, Rodriguez R (2018). Metformin reveals a mitochondrial copper addiction of mesenchymal cancer cells. PLoS One.

[55] Repiščák P, Erhardt S, Rena G, Paterson MJ (2014). Biomolecular mode of action of metformin in relation to its copper binding properties. Biochemistry.

[56] Holzer AK, Manorek GH, Howell SB (2006). Contribution of the major copper influx transporter CTR1 to the cellular accumulation of cisplatin, carboplatin, and oxaliplatin. Mol Pharmacol.

[57] Kuo Y, Gybina AA, Pyatskowit JW, Gitschier J, Prohaska JR (2006). Copper transport protein (Ctr1) levels in mice are tissue specific and dependent on copper status. J Nutr.

[58] Logie L, Harthill J, Patel K, Bacon S, Hamilton DL, Macrae K, McDougall G, Wang H, Xue L, Jiang H, Sakamoto K, Prescott AR, Rena G (2012). Cellular responses to the Metal-binding properties of metformin. Diabetes.

[59] Katano K, Safaei R, Samimi G, Holzer A, Rochdi M, Howell SB (2003). The copper export pump ATP7B modulates the cellular pharmacology of carboplatin in ovarian carcinoma cells. Mol Pharmacol.

[60] Fu S, Naing A, Fu C, Kuo MT, Kurzrock R (2012). Overcoming platinum resistance through the use of a copper-lowering agent. Mol Cancer Ther.

[61] Erices R, Bravo ML, Gonzalez P, Oliva B, Racordon D, Garrido M, Ibañez C, Kato S, Brañes J, Pizarro J, Barriga MI, Barra A, Bravo E, Alonso C, Bustamente E, Cuello MA, Owen GI (2013). Metformin, at concentrations corresponding to the treatment of diabetes, potentiates the cytotoxic effects of carboplatin in cultures of ovarian cancer cells. Reprod Sci.

[62] Wei D, Wang Y, Shi H (2018). Association of p53 and mitochondrial gene with chemosensitization by metformin in ovarian cancer. Oncotarget.

[63] Rizos CV, Elisaf MS (2013). Metformin and cancer. Eur J Pharmacol.

[64] Peppicelli S, Toti A, Giannoni E, Bianchini F, Margheri F, Del Rosso M, Calorini L (2016). Metformin is also effective on lactic acidosis-exposed melanoma cells switched to oxidative phosphorylation. Cell Cycle.

[65] Liu X, Romero IL, Litchfield LM, Lengyel E, Locasale JW (2016). Metformin targets central carbon metabolism and reveals mitochondrial requirements in human cancers. Cell Metab.

