# Supplementary material for: Phase I study of metformin in combination with carboplatin/paclitaxel chemotherapy in patients with advanced epithelial ovarian cancer
Source: Invest New Drugs. 2020 Mar 7;38(5):1454–62. doi: 10.1007/s10637-020-00920-7 (PMC7497683; doi:10.1007/s10637-020-00920-7)
Supplement: Supplementary file 1 — (DOCX 25 kb) [file 10637_2020_920_MOESM1_ESM.docx]

| **Supplementary Table 1**  In- and exclusion criteria | Inclusion criteria   - Advanced stage (FIGO III-IV), histologically confirmed and documented epithelial ovarian carcinoma - Patients eligible for neo-adjuvant carboplatin/paclitaxel chemotherapy prior to surgical debulking OR patients with relapsed or progressive ovarian cancer after initial treatment eligible for palliative carboplatin/paclitaxel chemotherapy - Eastern Cooperative Oncology Group-performance status (ECOG-PS) of 0-2 - Age ≥ 18 years - Laboratory Requirements - within 7 days prior to enrolment:   - absolute neutrophil count [ANC] ≥1.5 x 109/L   - platelets > 100 x 109/L   - hemoglobin >9g/dl. Patients may be transfused or use erythropoietin to maintain hemoglobin values ≥ 9 g/dl.   - hepatic function: bilirubin ≤1.5×upper limit of normal (ULN), AST/ALT≤2.5×ULN   - estimated creatinine clearance ≥ 60ml/min - Written informed consent according to ICH/GCP, and national/local regulations. |
| --- | --- |
|  | Exclusion criteria:   - Current or recent (within 30 days of first study dosing) treatment with another investigational drug or participation in another investigational study - Metformin within 4 weeks prior to enrolment - Symptomatic CNS metastasis - Pre-existing peripheral neuropathy ≥ CTC grade 2 - Pregnant or lactating females - Women of childbearing potential (defined as <2 years after last menstruation and not surgically sterile) not using effective, non-hormonal means of contraception (intrauterine contraceptive device, barrier method of contraception in conjunction with spermicidal jelly) during the study and for 6 months after the last study medication - Known hypersensitivity to any of the study drugs or excipients - Serious active infection requiring i.v. antibiotics at enrolment - Unstable medical conditions - Evidence of any other medical conditions, physical examination or laboratory findings that may interfere with the planned treatment, affect patient compliance or place the patient at high risk from treatment related complications |

| **Supplementary Table 2** Dose-limiting toxicities | Hematologic   - Absolute neutrophil count (ANC) < 0.5 x 109/L for at least 7 days - Febrile Neutropenia (ANC < 1.0 x 10^9^/L, fever > 38.5 ^0^C) - Platelets < 25 x 10^9^/L - Bleeding felt to be due to thrombocytopenia |
| --- | --- |
|  | Non-hematologic   - Diarrhea > Grade 3 despite optimal loperamide use persisting ≥ 2 weeks - Nausea / vomiting > grade 3 despite optimal use of anti-emetics, persisting ≥ 2 weeks or leading to > 7 missed doses of metformin. - Other grade 3 / 4 effects thought to be treatment related. - Missing >7 doses of metformin treatment for toxicity reasons. - Inability to administer next cycle of carboplatin/paclitaxel within 5 weeks after previous cycle |

| **Supplementary Table 3** Paclitaxel PK parameters | Parameter | Unit | Paclitaxel without metformin (median (IQR)) | Paclitaxel with metformin (median (IQR)) | p-value |
| --- | --- | --- | --- | --- | --- |
|  | CL | L/h | 26.6 (18.6) | 31.8 (5.6) | 0.286 |
|  | Vd | L | 642 (574) | 783 (543) | 0.374 |
|  | AUC 48 h | mg/L*h | 10.6 (8.6) | 8.96 (1.5) | 0.131 |
|  | AUC 48 h corrected for dose adjustment | mg/L*h | 10.6 (8.6) | 9.67 (1.6) | 0.131 |

Pharmacokinetic parameters of paclitaxel when administered as combination chemotherapy with carboplatin and with addition of metformin (n=11 patients). Paclitaxel AUC of cycle 2 was corrected for paclitaxel dose adjustment from cycle 1 to cycle 2 in 1 patient (dose reduction for reason of leukopenia), assuming a linear relationship between dose and AUC.

AUC area under the concentration-time curve, CL clearance, IQR inter quartile range, Vd total volume of distribution

| **Supplementary Table 4** Efficacy outcomes for the individual patients | Pt | Setting | Stage | Previous treatment lines | Cycles of combination therapy | Best overall response | Surgery | Time to relapse  (months) | Survival  (months) |
| --- | --- | --- | --- | --- | --- | --- | --- | --- | --- |
|  | 1 | Neoadjuvant | IV | 0 | 6 | PR | Optimal ID | 8 | 11 |
|  | 2 | Palliative | IIIC | 9 | 1 (+ 2 without metformin) | PD |  | 2 | 10 |
|  | 3 | Palliative | IV | 2 | 6 | PR |  | 35 | 44 |
|  | 4 | Palliative | IIIC | 4 | 6 | SD |  | 9 | 13 |
|  | 5 | Neoadjuvant | IV | 0 | 7 | PR | Optimal ID | 12 | 14 |
|  | 6 | Palliative | IV | 3 | 6 | PR |  | 10 | 30 |
|  | 7 | Palliative | IV | 1 | 6 | PR |  | 9 | 23 |
|  | 8 | Palliative | IV | 1 | 6 | PR |  | 7 | 8 |
|  | 9 | Neoadjuvant | IIIC | 0 | 6 | PR | Optimal ID | 21 | 28 |
|  | 10 | Palliative | IV | 2 | 6 | PR |  | 4 | 7 |
|  | 11 | Neoadjuvant | IV | 0 | 1 (+ 5 without metformin) | PR | Optimal ID | 21 |  |
|  | 12 | Palliative | IIIC | 1 | 1 (+ 4 without metformin) | PR |  | 12 | 30 |
|  | 13 | Neoadjuvant | IIIC | 2 | 4 | PR | Complete ID | 34 |  |
|  | 14 | Palliative | IIIC | 1 | 6 | PR |  | 5 | 9 |
|  | 15 | Palliative | IIIC | 1 | 8 | PR |  | 10 | 25 |

Efficacy parameters for the individual patients. Time to symptomatic relapse was calculated from the first day of the first study treatment cycle to the day of first documentation of symptomatic progression. Analysis of response and follow-up for progression of disease was not standardized but was performed according to standard clinical practice. Survival was calculated from the first day of the first study treatment cycle to the day of death.

PD progressive disease, PR partial response, ID interval debulking surgery

| **Supplementary Table 5** Early phase trials of metformin in combination with systemic anti-cancer therapies | Year | Author | Disease | Systemic treatment | Metformin daily dose | DLT/AE leading to dose reductions | Dose escalation scheme |
| --- | --- | --- | --- | --- | --- | --- | --- |
|  | 2015 | Sayed | Stage IV NSCLC | Cisplatin + gemcitabine | 500 mg | None | N |
|  | 2018 | Molenaar | Advanced refractory cancers | Everolimus | 1000 mg | Several; no safe RP2D | Y |
|  | 2018 | Trucco | Pediatric ALL | Vincristine, dexamethasone, PEG-asparaginase, doxorubicin | 1000 mg/m^2^ | Diarrhea | Y |
|  | 2017 | Morgillo | Stage IV NSCLC | Erlotinib | 1500 mg | GI | Y |
|  | 2016 | Miranda | Refractory mCRC | 5-FU | 1700 mg | GI | N |
|  | 2019 | Yam | HR+ MBC | Everolimus + exemestane | 2000 mg | None | Y |
|  | 2016 | Khawaja | Advanced refractory cancers | Temsirolimus | 2000 mg | Mucositis | Y |
|  | 2019 | Nanni | MBC | Liposomal doxorubicin + cyclophosphamide | 2000 mg | None | ? |
|  | 2017 | Parikh | Advanced NSCLC | Carboplatin + pemetrexed | 2000 mg | None | Y |
|  | 2016 | Reni | Metastatic pancreatic cancer | Cisplatin, epirubicin, capecitabine, gemcitabine | 2000 mg | None | N |
|  | 2015 | Kordes | Metastatic pancreatic cancer | Erlotinib + gemcitabine | 2000 mg | GI | Y |
|  | 2018 | Marrone | Metastatic non-squamous NSCLC | Carboplatin, paclitaxel, bevacizumab | 2000 mg | None; closed prematurely due to slow accrual | ? |
|  | 2018 | Ramos | ALL | Prednisone; pre-induction to chemotherapy | 2550 mg | Not reported | N |

AE adverse event, ALL acute lymphoblastic leukemia, CRC colorectal cancer, DLT dose-limiting toxicity, GI gastro-intestinal, HR hormone receptor, MBC metastatic breast cancer, NSCLC non-small cell lung cancer, RP2D recommended phase II dose.
